# Remaining Useful Life Estimation of Hollow Worn Railway Vehicle Wheels via On-Board Random Vibration-Based Wheel Tread Depth Estimation

**DOI:** 10.3390/s24020375

**Published:** 2024-01-08

**Authors:** Ilias A. Iliopoulos, John S. Sakellariou

**Affiliations:** Stochastic Mechanical Systems & Automation (SMSA) Laboratory, Department of Mechanical Engineering and Aeronautics, University of Patras, 26504 Patras, Greece; ilias.iliopoulos@ac.upatras.gr

**Keywords:** on-board measurements, vibration signals, accelerometer, remaining useful life estimation, railway vehicles, hollow worn wheels, statistical time series methods, stochastic modeling

## Abstract

The problem of remaining useful life estimation (RULE) of hollow worn railway vehicle wheels in terms of remaining mileage via wheel tread depth estimation using on-board vibration signals from a single accelerometer on the bogie frame is presently investigated. This is achieved based on the introduction of a statistical time series method that employs: (i) advanced data-driven stochastic Functionally Pooled models for the modeling of the vehicle dynamics under different wheel tread depths in a range of interest until a critical limit, as well as tread depth estimation through a proper optimization procedure, and (ii) a wheel tread depth evolution function with respect to the vehicle running mileage that interconnects the estimated hollow wear with the remaining useful mileage. The method’s RULE performance is investigated via hundreds of Simpack-based Monte Carlo simulations with an Attiko Metro S.A. vehicle and many hollow worn wheels scenarios which are not used for the method’s training. The obtained results indicate the accurate estimation of the wheels tread depth with a mean absolute error of ∼0.07 mm that leads to a corresponding small error of ∼3% with respect to the wheels remaining useful mileage. In addition, the comparison with a recently introduced Multiple Model (MM)-based multi-health state classification method for RULE, demonstrates the better performance of the postulated method that achieves 81.17% True Positive Rate (TPR) which is significantly higher than the 45.44% of the MM method.

## 1. Introduction

Hollow wear constitutes the predominant degradation of tread on railway vehicle wheels, inducing a substantial impact on the vehicle’s steering precision and stability, especially once a critical limit of the wheel tread depth (typically ≲2.5–3.0 mm) is exceeded. This is due to the resultant significant lateral vibrations that may lead to adverse consequences such as passenger discomfort, damage in the neighboring rolling stock, degradation of the track infrastructure, or, in the worst case, to derailment [1,2,3,4,5]. Therefore, the automated and prompt determination of the remaining useful mileage, or else the *remaining useful life estimation (RULE)*, of railway vehicle wheels with hollow wear, before the critical limit is reached, via on-board vibration response signals, is imperative. In addition, wheels RULE may play a vital role in the broader Condition-Based Maintenance (CBM) management of railway vehicles, as it may lead to enhanced comfort, safety and reliability of the vehicles, as well as to reduced maintenance cost and downtime.

In a broader context, vibration-based RULE technology has witnessed rapid advancements over the previous two decades, yielding a wealth of literature and applications at both component and system level [6,7,8,9,10]. Vibration-based RULE is typically pursued via two main data-driven types of methods: a more commonly used one that addresses RULE as a *prediction problem*, and another that treats it as a *multi-health state classification* problem.

Despite the significant progress of vibration-based RULE, related applications for automated RULE in railway vehicle wheels using on-board measurements remain scarce [11,12,13,14], while the considered wheels possess severe faults in their treads, such as cracks and spalling. The methods of these studies fall under the category of the prediction-based RULE with the majority relying on statistical models, which incorporate a Wiener process model integrated with a linear, power-law, or exponential deterministic function according to the considered defect [11,12,13], whereas only the study in [14] utilizes an AI/ML model via the Neural Basis Expansion Analysis for Time Series (N-BEATS). The vibration signals RMS is employed as the selected feature for RULE in [11,12,13], where statistical models are also employed and the obtained results indicate adequate agreement between the RMS evolution and the selected deterministic function representing wheel degradation. However, these studies explore quite severe wheel defects, as previously mentioned, implying RULE at the latest level of wheels’ lifetimes, while the RMS user selected threshold corresponding to the end-of-life is subjectively selected and may not always coincide with the actual failure event of the railway wheels, thus jeopardizing the vehicle’s safety.

On the other hand, RULE in [14] is performed for a wide mileage range via the N–BEATS predictor and the use of the vibration signals variance after Box–Cox transformation as the selected feature. This transformation is employed to minimize time-varying effects on the vibration signals, with thousands of them to be used for the training (hyperparameters determination) of the N-BEATS predictor. In contrast to previous studies, the problem pertaining to the assumption that the wheel failure event occurs when the selected RULE feature exceeds a subjectively pre-specified threshold is acknowledged and addressed via multiple user-specified thresholds. The risk of inaccurate RULE is mitigated with this procedure, yet the risk for poor estimates still exists as the selection of these thresholds remains subjective. It is also worth mentioning that the methods in all the above studies use vibration measurements obtained from the vehicle axlebox, necessitating the frequent calibration and potential replacement of the employed sensors due to the extremely harsh operating conditions [15].

An alternative method [16] that has been recently introduced by the authors and collaborators tackles the RULE problem of hollow worn railway vehicle wheels as a multi-health state classification problem [17,18,19,20] demonstrating a promising performance. The method presents high sensitivity to the subtle effects of the hollow worn wheels on the vehicular dynamics, yielding from the fact that the Power Spectral Density obtained from acceleration signals on the vehicle bogie is employed as the main feature for the classification procedure, providing comprehensive information on the vehicular dynamics compared with static quantities such as the vibration signals RMS, the variance, and so on. However, the typical issue of the multi-health state classification methods pertaining to the classification of an unknown state into more than one relatively close health states has not been overcome.

The goal of this study is the introduction and assessment of a statistical time series method for automated remaining useful life estimation of hollow worn railway vehicle wheels via on-board precise tread depth estimation using lateral vibration signals from a single accelerometer on the bogie frame. The method is based on: (i) A Functionally Pooled AutoRegressive (FP–AR) model that belongs to the broader family of the advanced data-driven Functional Models [21]. This model is employed for the representation of the partial vehicular dynamics for different hollow wear levels within a continuous tread depth range and until a critical limit, as well as for tread depth estimation through a proper optimization procedure; and (ii) a wheel tread depth evolution function with respect to the vehicle running mileage that interconnects the estimated tread depth (hollow wear) with the remaining useful mileage.

The FP–AR model is estimated in a *baseline (training) phase* via a limited number of vibration signals obtained from a single accelerometer on the bogie of the traveling vehicle, for a sample of known wheels tread depths within the range of interest at the corresponding running mileage. The collection of the baseline phase signals and the associated tread depth values is carried out at known periodic running mileage intervals during an initial wheels re-profiling cycle (operational lifespan in terms of mileage) that ends when the tread depth approaches a critical limit (≤2.5 mm) and new wheel re-profiling is necessary [4]. Once the initial re-profiling cycle has been completed, an evolution function that maps the tread depth to the running mileage is also constructed via conventional fitting approaches, using the available running mileage and the corresponding tread depth values. With the completion of these steps of the method’s training, it may operate continuously or on demand in real time (*inspection phase*) for the RULE of wheels under unknown hollow wear using a fresh vibration signal that is driven through the FP–AR model for precise wheel tread depth estimation via Nonlinear Least Squares optimization. The obtained tread depth is then fed into the inverse evolution function and the remaining useful life of the hollow worn railway wheels in terms of remaining mileage is estimated.

Based on all the above, the novel contributions of the present study are summarized as follows:

- The *automated* RULE of hollow worn railway wheels via their on-board tread depth estimation is for the first time achieved using a *limited* number of vibration signals for the training of the employed method, which are obtained from a *single* accelerometer located on the vehicle bogie avoiding thus the harsh environment of the axlebox area.
- The on-board wheels tread depth estimation is based on the advanced modeling provided by the FP–AR model, which may account for the complete information—there is no need for predefined frequencies or other static quantities (e.g., RMS, kurtosis, etc.) with limited information—of the vehicle dynamics that is included in the vibration signals, leading thus to the *precise* (no gross classification) estimation of the wheels tread and thus their RULE. Based on this, the introduced method is characterized by high *sensitivity* to any subtle change to the wheels tread achieving so *prompt* RULE of hollow worn wheels, as it is the case of wheels with approximately zero tread depth.
- The use of the wheel tread depth as the feature that leads to the estimation of their remaining mileage is a unique characteristic of the introduced method, due to the fact that the tread depth is the *inherent quantity* that characterizes hollow worn wheels, always with a monotonic pattern that facilitates prognostics. Additionally, tread depth provides *valuable* insight for maintenance as one of the most important wheels characteristics that determines their remaining useful life.

The postulated method is assessed via 566 Monte Carlo simulations using the well-known Simpack software 2019.1 [22] for the development of a 42 degrees-of-freedom model representing an Attiko Metro S.A. railway vehicle running over a straight track with speed equal to 35 m/s. A single vibration signal is obtained from an accelerometer on the vehicle’s bogie in each simulation, while a wide range of railway wheel tread depths of hollow worn wheels is covered within the range [0,2.3] mm using actual measurements obtained from the Attiko Metro S.A. maintenance operator (Athens, Greece). In addition, the introduced method is compared with the multi-health state classification RULE method of [16], which is based on multiple models for the representation of various hollow wear levels corresponding to distinct tread depth ranges. The selection of this recently introduced method is based on its promising results, which are presented in [14], while it is also the only available vibration-based method that employs measurements of wheel tread depth for the estimation of their remaining mileage that will allow a fair comparison with the method presented in this study.

The rest of this article is organized as follows: The operating framework is presented in Section 2. The Monte Carlo simulations via a Simpack-based railway vehicle model are presented in Section 3, while the remaining useful life estimation method is presented in Section 4. The performance assessment of the RULE method is presented in Section 5, whereas the comparison with a multi-health state classification method for RULE in Section 6. Finally, a discussion and the concluding remarks of the study are summarized in Section 7.

## 2. The Operating Framework

**Figure 1:**
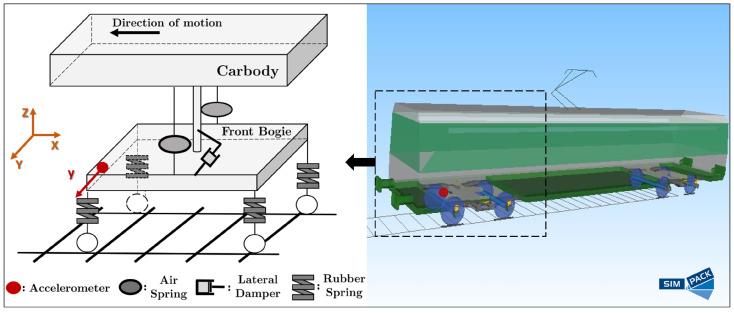
Schematic of the Simpack-based railway vehicle model including the employed accelerometer location on the bogie frame.

This section describes the operating framework of the presented method for hollow wheels RULE including all necessary prerequisites for the method’s proper training and real time operation using on-board lateral vibration signals from a single accelerometer on the railway vehicle bogie frame. The framework considers a railway vehicle traveling with constant speed and payload under normal operation over a straight track, and consists of two main phases (see details in Section 4):

- *Baseline phase:* This is the training phase of the method which is performed once in an initial wheels re-profiling cycle where *M* measurements of random vibration acceleration signals for a sample of distinct, running mileages $\tau_{i}$ ( i=1,…,M ) along with the associated wheels tread depths $k_{i}$ are performed. A *N*-sample-long vibration acceleration signal $y_{k}\left[t\right]$ ( t=1,…,N designating the normalized with the sampling period discrete time) is obtained from the vehicle’s bogie (Figure 1) in each measurement and thus a total of *M* vibration signals are available. It is noted that measurements are performed for tread depths in the range of $\left[0,k_{max}\right]$ , where $k_{max}$ (herein ≤2.5 mm) is a critical limit that is defined by the railway vehicle maintenance operator. In addition, tread depths are considered only for the leading vehicle wheels expecting similar wear for the following wheels, according to Attiko Metro S.A. maintenance experts.
- *Inspection phase:* This phase is performed in real time (new re-profiling cycle) once the training of the RULE method has been completed, using an on-board measurement of a new acceleration signal $y_{u}\left[t\right]$ from the accelerometer location used in the baseline phase, corresponding to unknown (subscript ‘u’ designates unknown) wheels tread depth or else unknown *k*, and thus unknown wheel remaining useful mileage to be estimated with the method’s operation.

## 3. The Monte Carlo Simulations via a Simpack-Based Railway Vehicle Model

### 3.1. The Railway Vehicle Model

The Monte Carlo simulations are based on a detailed 42-DOF Simpack-based [22] multibody model (Figure 1) that has been developed for the dynamics representation of a trailing railway vehicle. This model includes 15 distinct rigid components: four wheelsets consisting of eight axle-boxes, two bogie frames, and the car body. Each simulation run has been performed using a different straight track segments with track irregularities conforming to the ERRI B176 standard [23]. The geometric and suspension parameters of this railway vehicle model are derived from a standard trailing car of a third generation vehicle used by the Attiko Metro S.A.; see details in [4].

### 3.2. The Hollow Wear Conditions, the Simulations and the Vibration Signals

The Monte Carlo simulations with the hollow wheels are based on actual tread depth measurements given by the Urban Rail Transport S.A., which is the maintenance operator of the Attiko Metro S.A. vehicles, throughout a wheels re-profiling cycle for a passenger vehicle that ends when the tread depth reaches the critical value of ∼2.5 mm. The actual tread depths along with the corresponding running mileage are presented in Table 1. Based on the fact that these measurements are very limited for a statistically reliable assessment of the hollow wheels RULE method that is presented in this study, a typical exponential function has been developed using the values of Table 1 through typical curve fitting via the exponential curve of the form: $k=ae^{b\tau }-a$ ( a=1.80 and $b=3.33\times 10^{-6}$ ) achieving $R^{2}=98.45\%$ goodness-of-fit (Matlab function: fit.m) for the representation of the tread depth evolution with respect to mileage increase.

Based on this, 137 tread depths at corresponding mileages are used in the Simpack-based model for distinct simulations and generation of acceleration signals at the vehicle bogie (see Figure 1).

A single lateral vibration signal from the accelerometer on the bogie frame (Figure 1) with the vehicle traveling at v=35 m/s is obtained per simulation, sampled at fs=150 Hz (acceleration signal bandwidth [0–75] Hz) with length N=3600 samples (24 s); see also Table 2.

A total of 566 simulations are conducted with the vehicle running on a different straight track segment in each simulation, with acceleration signals from only 18 simulations from an initial wheels re-profiling cycle to be used for the training of the RULE method in the baseline phase. These correspond to a sample of distinct tread depths in the range of [0,2.3] mm measured at a mileage interval of 14,400 km, which is a realistic condition for standard wheels tread inspection. The remaining 548 simulations are solely used for the thorough assessment of the RULE method in the inspection (online) phase. These correspond to four acceleration signal sets obtained from four distinct re-profiling cycles, each one including 137 tread depths (wheels hollow wear), which are obtained due to mileage increase with an of interval of 1800 km within the considered range of [0, 244,800] km. All details on the considered wheel hollow wear (HW) in terms of tread depths which are used in the simulations, as well as about the employed vibration signals, are presented in Table 2.

### 3.3. The Hollow Worn Wheels and Their Effects on the Vibration Signals

**Figure 2:**
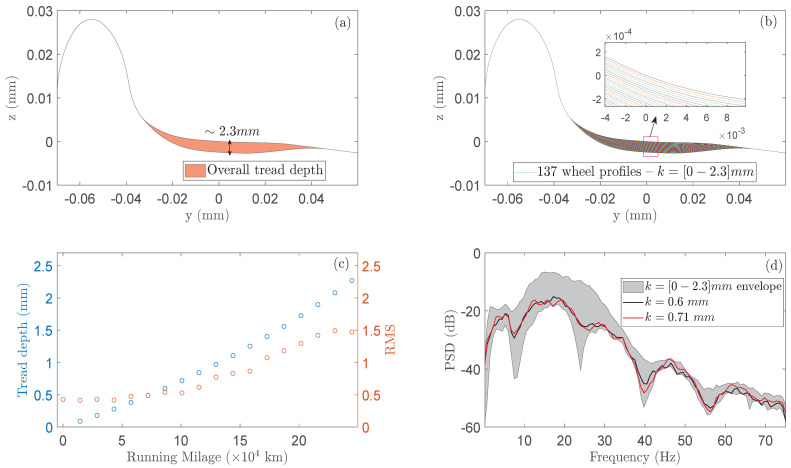
(**a**) Visual interpretation of the considered hollow wear on the wheels profile. (**b**) The 137 wheel profiles which are considered in the Monte Carlo simulations. (**c**) The tread depths used in the baseline phase versus the corresponding vibration signals RMS values with respect to running mileage. (**d**) PSD envelope estimated using the Welch method and the acceleration signals corresponding to the 18 tread depths of the baseline phase along with two individual PSD estimates for two adjacent tread depths.

The visual interpretation of the considered railway wheel tread depths in the range of [0, 2.3] mm are illustrated in Figure 2a,b. In particular, Figure 2a depicts the wheels wear for the tread depth critical limit of 2.3 mm, where the wheels should be reprofiled for subsequent use, while Figure 2b depicts the various stages (137 tread depths) of the wheels wear until the critical limit. These profiles have been constructed using the actual tread depths, which are mentioned in the previous subsection, along with related wheels dimensions (i.e., Sd, Sh, qR, Tw) as described in [5], for their implementation in the Simpack-based Monte Carlo simulations.

On the other hand, Figure 2c depicts the increasing pattern of the 18 tread depths (left y axis) which are used for the training of the RULE method along with the RMS values (right y axis) from the corresponding acceleration signals (per tread depth) with respect to the increasing running mileage (x axis). As is evident, the RMS values do not exhibit a consistently increasing pattern, similar to that of the tread depth (hollow wear), indicating that the selection of the RMS as a feature for railway vehicle RULE is inappropriate. Furthermore, Figure 2d illustrates Welch-based PSD estimates (Welch estimation details: Matlab function pwelch.m , Hamming window, window length = 256 Hz, overlap = 90%, frequency resolution df=0.586 Hz) ([24] p. 186) for two distinct tread depths, specifically 0.60 mm and 0.71 mm, as well as for the whole tread depth range. On the one hand, this graph indicates the substantial impact of wheels hollow wear on the vehicle dynamics, while on the other, that the effects of two close tread depths, such as the 0.60 mm (at 72,000 km) and 0.71 mm (at 86,400 km), are very similar underscoring the challenge for precise on-board tread depth estimation and thus wheels RULE in such scenarios.

## 4. The Remaining Useful Life Estimation Method

The RULE method of the present study is based on two core components: (i) a special form of data driven stochastic models, *the Functionally Pooled (FP) models* [21], which offer the capability of representing the vehicular dynamics for different depths of wheel tread in a continuous range of interest, and (ii) a *unique RULE concept* incorporating on-board tread depth estimation through a proper FP model motivated by [25] and the *mapping* of the obtained estimate through a proper evolution function to a running mileage according to the wheel’s hollow wear, which does not necessarily coincide with the current mileage of the train speed meter. As also mentioned in Section 2, the RULE method consists of a baseline (training) phase that is performed in an initial re-profiling cycle of the wheels using a number of *M* vibration signals obtained from an accelerometer on the bogie frame, each one corresponding to a known running mileage $\tau_{i}$ and tread depth $k_{i}$ ( i=1,…,M ), as well as an inspection phase, which may run on-demand or continuously during the railway vehicle normal operation (online) based on acquired vibration signals from the vehicle traveling with wheels under unknown hollow wear (tread depth), for which the RULE should be achieved.

In particular, the modeling of the partial vehicular dynamics in *the baseline phase* is performed through the estimation of a Functionally Pooled AutoRegressive (FP–AR) model of the form [21]:

(1) $$\begin{array}{c} y_{k}\left[t\right]\;+\sum_{i=1}^{na}a_{i}\left(k\right)\cdot y_{k}\left[t-i\right]=e_{k}\left[t\right],\;e_{k}\left[t\right]∼\mathrm{NID}\left(0,\;\sigma_{e}^{2}\left(k\right)\right)\;\mathrm{with}\;k\in R\;\\ a_{i}\left(k\right)=\sum_{j=1}^{p}a_{i,j}\cdot G_{j}\left(k\right) \end{array}$$

with na designating the AR order, $e_{k}\left[t\right]$ the model residual signal and NID (·,·) Normally Independently Distributed with the indicated mean and variance. The AR parameters $a_{i}\left(k\right)$ are modeled as explicit functions of tread depth *k* using a *p*-dimensional functional subspace spanned by the mutually independent functions $G_{1}\left(k\right),\ldots ,G_{p}\left(k\right)$ , while the constants $a_{i,j}$ designate the corresponding AR projection coefficients. The FP–AR model identification is based on [21]: (i) the determination of the FP–AR model order na and its functional subspace dimensionality *p* for a given basis function family (any type of orthogonal polynomials such as Legendre or Chebyshev may be equivalently used) using a Genetic Algorithm (GA) for the minimization of the Bayesian Information Criterion (BIC); (ii) the model estimation based on Ordinary Least Squares (OLS) using “data pooling” for the *M* available vibration signals (see also Section 2) corresponding to the sample of known tread depths $k_{1},\ldots ,k_{M}$ within the range of interest $\left[0,k_{max}\right]$ , where typically $k_{max}=k_{M}$ ; and (iii) the model validation via testing of the obtained model residual signals the whiteness (uncorrelatedness) hypothesis. Additionally, this phase includes the development of a tread depth *evolution function* k=f(τ) that interconnects the available tread depth samples with the corresponding mileage through typical curve fitting approaches. It should be noted that this function is similar with the one which, by exception, is formulated in this study, (Section 3) for the generation of a significant volume of data due to the limited actual measurements, thus allowing the method’s statistical reliable assessment, yet this will not be the case in an actual application of the method.

In the *inspection phase*, a vibration signal $y_{u}\left[t\right]$ (subscript *u* stands for unknown) is acquired from the vehicle traveling in a new re-profiling cycle under potentially hollow worn wheels with unknown tread depth *k* (also see Section 2), and the railway wheels RULE method is activated through the following two steps:

**Step 1:**  Given the estimated FP–AR( ${\left(na\right)}_{p}$ model of the baseline phase, $y_{u}\left[t\right]$ is substituted in Equation (1), the latter is solved with respect to $e_{u}\left[t,k\right]$ and based on this expression the estimation of the unknown *k* (current tread depth) is achieved using the following Nonlinear Least Squares (NLS) estimator, which is implemented by golden search and parabolic interpolation [25]:
(2) $$\hat{k}=\arg\min_{k}\sum_{t=1}^{N}e_{u}^{2}\left[t,k\right]$$
  with $\hat{\;}$ designating the estimate and $e_{u}\left[t,k\right]$ the model residuals under an unknown tread depth. The obtained $\hat{k}$ is finally validated through typical hypothesis testing of $e_{u}\left[t,k\right]$ whiteness via the Pena–Rodriguez test statistic *D*, which follows a standard normal distribution for a white sequence [26]. The whiteness hypothesis is accepted *iff* |D|≤Z with *Z* indicating a user selected threshold that is determined using typical normal distribution risk levels or heuristically based on the max absolute value of *D* as obtained from the identified FP–AR model residual signals. The successful validation of the $\hat{k}$ estimate indicates that the current dynamics as obtained from the measured vibration signal $y_{u}\left[t\right]$ may be accurately represented by the FP–AR model of the baseline phase and the estimate $\hat{k}$ of the tread depth is accepted.
**Step 2:**  Based on the tread depth evolution function of the baseline phase, the indicated hollow wear by $\hat{k}$ is matched with the corresponding running mileage ${\hat{\tau }}_{u}$ that leads to such a hollow wear which, as mentioned previously, may not necessarily be the same with the current mileage indicated by the train speed meter. Thus:
(3) $${\hat{\tau }}_{u}=f^{-1}\left(\hat{k}\right)$$
  and wheels RULE in terms of wheels remaining mileage ${\hat{\tau }}_{u}^{r}$ estimation is readily obtained as:
(4) $${\hat{\tau }}_{u}^{r}=\tau_{M}-{\hat{\tau }}_{u}$$
  with $\tau_{M}$ indicating the running mileage corresponding to the selected critical limit of the tread depth $k_{M}$ as defined in the baseline phase.

## 5. Performance Assessment of the Remaining Useful Life Estimation Method

*Baseline (training) phase*: The M=18 acceleration signals from the initial re-profiling cycle, as obtained from the accelerometer on the vehicle bogie (also see Table 2), with each one corresponding to a known tread depth $k_{i}$ and running mileage $\tau_{i}$ ( i=1,…,18 ), are used for the identification of an FP–AR(62)_4_ model with order na=62 and functional subspace consisting of p=4 Shifted Legendre polynomials; see estimation details in Table 3). This model represents the partial lateral vehicular dynamics for any wheel tread depth lying within the continuous range of [0,2.3] mm. Furthermore, the samples of the actual tread depths and corresponding mileages (Table 1) are used for the determination of the tread depth evolution function, which is of the exponential form (as made for Section 3): $k=ae^{b\tau }-a$ with all details given in Table 3 (Baseline phase).

**Figure 3:**
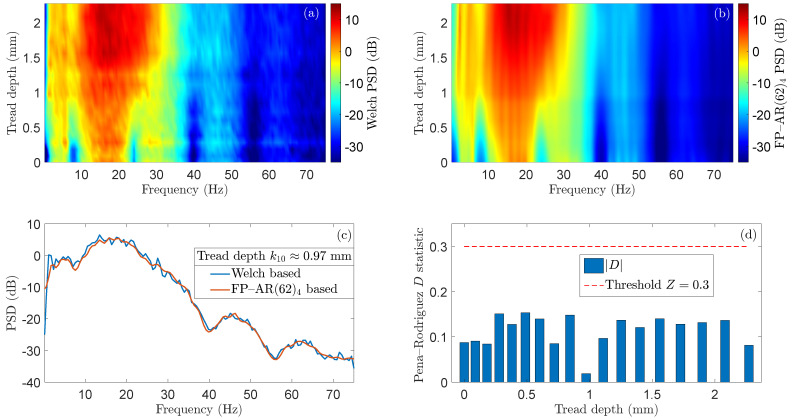
FP–AR(62)_4_ model validation results (Baseline phase): (**a**) Welch-based and (**b**) FP–AR(62)_4_-based PSD estimates using the 18 acceleration signals (initial re-profiling cycle) of the baseline phase. (**c**) Indicative Welch- and FP–AR(62)_4_-based PSD estimates for tread depth $k_{10}\approx 0.97$ mm. (**d**) FP–AR(62)_4_ model residual whiteness test results in terms of the Pena–Rodriquez *D* statistic along with the user selected threshold.

Figure 3a,b depicts the excellent agreement of the PSD with respect to the wheels tread depth as obtained by the FP–AR(62)_4_ and its corresponding Welch-based counterpart, indicating the FP–AR model’s capability to represent the vehicle dynamics for varying tread depths with high accuracy. Figure 3c includes a similar comparison for one indicative test case corresponding to a specific tread depth where the accurate modeling is also evident. Figure 3d depicts the values of the *D* statistic as obtained from the Pena–Rodriguez whiteness test based on the 18 model residual (error) signals as they are obtained from the estimated FP–AR (62)_4_ model, as well as the threshold Z=0.3 which is heuristically selected to be almost two times higher than the maximum absolute value of *D*.

*Inspection (online) phase*: The 4 signal sets of Table 2 that lead to the 548 test cases with hollow worn wheels are used in this phase for the method’s RULE performance assessment. It is worth noting that in all these test cases the wheels wear (tread depth) is unknown and their remaining useful mileage should be estimated. The available vibration acceleration signal from each test case is driven through the FP–AR(62)_4_ model and the wheels tread depth $\hat{k}$ along with the remaining mileage $\tau_{u}^{r}$ are estimated via Equations (2) and (4), respectively.

**Figure 4:**
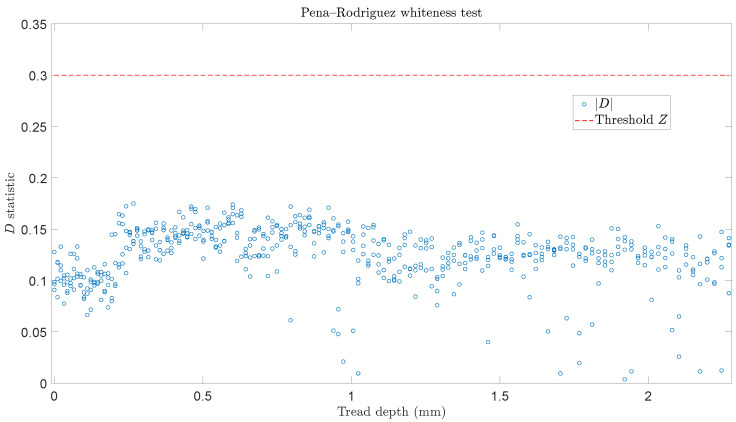
Validation of the FP–AR(62)_4_-based tread depth estimates (Inspection phase): an estimate is validated if the Pena–Rodriquez *D* statistic is under the user selected threshold (548 signals in total, 4 signal sets with each set corresponding to 137 tread depths).

Figure 4 depicts the scatter plot of the method’s *D* statistical values for all considered tread depths corresponding to the 548 signals of the inspection phase, as well as the threshold of *D* ( Z=0.3 ) as selected in the baseline phase. As is evident, all *D* values are under the threshold indicating that all tread depth estimates through the FP–AR(62)_4_ model are valid, and may be used in the evolution function for the wheels remaining useful mileage estimation. The remarkably low estimation mean errors of wheel tread depths for each signal set are presented in Table 4, indicating the precise on-board estimation of wheels wear based on the employed FP–AR model.

**Figure 5:**
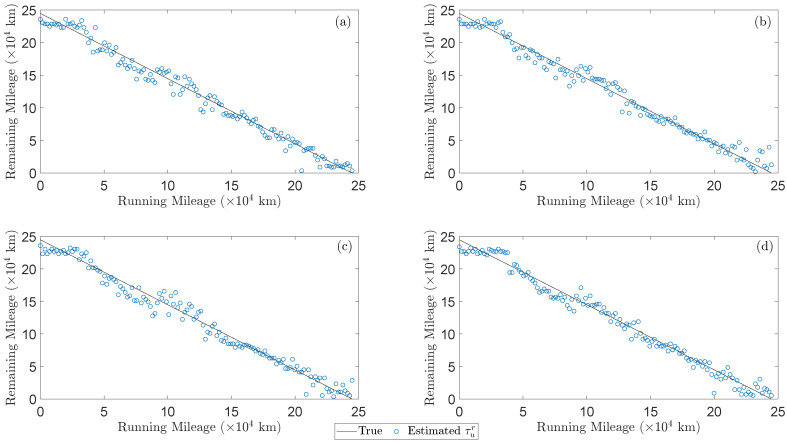
Hollow worn wheels remaining useful life estimation results (Inspection phase). Each subplot includes remaining mileage estimates (blue circles) for the 137 test cases of hollow worn wheels along with the true mileage (black line) using the acceleration signals from: (**a**) Set 1, (**b**) Set 2, (**c**) Set 3, (**d**) Set 4.

Once the precise estimates of the wheels tread depths that are under unknown hollow wear are validated, their remaining mileage is obtained using the inverse evolution function, which is shown in Table 3 (see inspection phase), and the results for the 548 test cases are presented in Figure 5. In particular, each subplot includes the remaining mileage estimates (blue circles) as obtained by the method based on the signals from one of the four considered signal sets corresponding to 137 distinct tread depths (see Table 2) along with the actual values (black line). As it is evident, the method is capable of estimating accurately the wheels remaining useful life from the beginning of the vehicle operation (re-profiling cycle), which is also confirmed by the the Mean Absolute Error (MAE) that is presented in Table 4 per signal set.

## 6. Comparison with a Multi-Health State Classification Method for RULE

The comparison of the postulated method with a multi-health state classification-based RULE method, which is based on multiple, conventional, AutoRegressive models (MM–AR), is presented in this section. A non–parametric variant of this method with very promising results has been recently presented by the authors and collaborators of [16].

For a fair comparison, the railway wheels HW for all considered re-profiling cycles is divided into 7 distinct “health states” associated with specific railway wheel tread depth ranges and remaining mileage intervals as shown in Table 5. Based on these, the comparison between the two methods is performed according to their capability to correctly classify the unknown tread depths to the specific health states, which as mentioned in the introduction corresponds to a gross and not precise RULE.

There is no new training procedure for the FP–AR-based method, while the same AR order ( na=62 ) is adopted for the MM–AR-based method. However, the latter needs 56 signals (8 acceleration signals per health state) from the initial re-profiling cycle for its training, in contrast to the FP–AR, which is trained with M=18 vibration signals. Thus, both methods’ assessment is performed in this section using 510 vibration signals. The parameter vectors of the multiple AR models is the feature that is employed by the MM–AR-based method for RULE, which is achieved through multi-health state classification. The decision mechanism is based on the Mahalanobis distance between the available baseline phase model parameter vectors (8 per health state) and is obtained from a single AR model of the same order estimated in the inspection phase for each of the considered 510 test cases. On the other hand, there is no decision making mechanism for the FP–AR-based method and just the estimated remaining mileage ${\hat{\tau }}_{u}^{r}$ is classified into one of the remaining mileage ranges (Table 5) and thus to the corresponding wheels health state.

**Figure 6:**
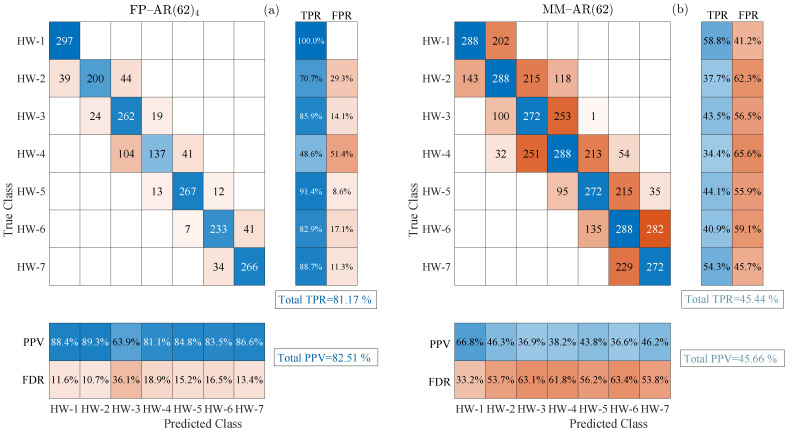
Confusion matrices including the multi-health state classification-based RULE results for: (**a**) the FP–AR(62)_4_-based method and (**b**) the MM–AR(62)-based method. Blue tint: correct classification, red tint: misclassification. (2040 inspection test cases per method).

An S-fold cross validation procedure ([27] p. 33) is adopted for the methods’ thorough assessment and fair comparison including “rotation” of the signals, which are used in the baseline phase for the training of the methods. In particular, four rotations are performed per method, leading thus to a total of 2040 ( 4×510 ) inspection cases. The classification-based RULE results for both methods are presented via confusion matrices [28] in Figure 6. The FP–AR-based method achieves much higher correct classification percentages in all considered inspection cases than the MM–AR-based method, although it is not designed for such type of operation.

In each confusion matrix, the blue colored diagonal and the red colored off-diagonal cells correspond to the number of correctly and incorrectly classified inspection cases, respectively. The row summaries on the right of each matrix indicate the percentages of correctly (True Positive Rate or TPR) and incorrectly (False Positive Rate or FPR) classified inspection cases per true health state with blue and red tint, respectively [28]. On the other hand, column summaries at the bottom of each display the Positive Predictive Values (also referred to as PPV or precision) and False Discovery Rates (also known as FDR or 1 − precision) per predicted health state using the same color code as previously [28].

The overall percentages of the correctly classified inspection cases (True Positive Rate) and Positive Predictive Values (precision) are summarized in Table 6. Based on these, the superiority of the proposed method, even if it is used as classification-based RULE is evident reaching an overall 81.17% true positive rate and 82.51% precision, while the performance of the MM–AR-based method is poor achieving 45.44% and 45.66% , respectively.

## 7. Conclusions and Discussion

The problem of automated remaining useful life estimation (RULE) of hollow worn railway vehicle wheels via on-board precise wheel tread depth estimation using lateral vibration signals from a single accelerometer on the bogie frame has been addressed has been, for the first time, addressed in this study, based on a a statistical time series method. This is a problem of high importance, as hollow worn wheels lead to significant lateral vibrations of the vehicle, and the prompt determination of their remaining useful mileage (or else the RULE), before the critical limit is surpassed, may reduce maintenance cost and enhance passenger comfort and safety.

The introduced method is founded on two main components: (a) an FP–AR model for the vehicular dynamics representation and wheels precise tread estimation, and (b) a wheel tread depth evolution function interconnecting the estimated tread depth (hollow wear) with the corresponding remaining mileage. In particular, an FP–AR(62)_4_ model for the representation of the vehicular dynamics within the continuous tread depth range [0,2.3] mm and an exponential function for the tread depth evolution with respect to the vehicle running mileage have been employed. The training of the method has been based on a limited number of 18 acceleration signals from an initial wheel re-profiling cycle, while its performance has been assessed using vibration signals from four more, distinct, re-profiling cycles that led to 548 inspection test cases with hollow worn wheels. Furthermore, a comparison with an MM–AR-based method in a multi-health state classification framework for RULE has been performed based on 2040 inspection cases.

Based on the results of Section 5, as well as the pertinent state of the art on the topic that is presented in Section 1, the main lessons learned from the study are concisely summarized and discussed below:

(i) A key advantage of the proposed method is the fact that the wheel RULE is achieved using a very special feature, the wheel tread depth which, unlike with the statistical models—which rely on static quantities such as the RMS, kurtosis and thus on exploiting limited information of the vehicle dynamics—is the characteristic that describes exactly the considered hollow wear, and is precisely estimated through the FP–AR model using the full information included in the vibration signals. Another advantage of the introduced method is that its training requires only a limited number of on-board vibration signals (18 are used in this study) from a single re-profiling cycle, compared to the AI/ML model-based methods that typically require a significant number of vibration signals from multiple run-to-failure experiments. Regarding the multi-class classification methods, the advantage of the new method is its capability to provide precise RULE instead of rough classification.
(ii) Other general advantages of the introduced method that may be mentioned are: (a) Its capability to operate with just a single accelerometer placed on the vehicle’s bogie frame that effectively circumvents the problem of frequent calibration or even replacement of sensors which are commonly installed to the axlebox area with highly harsh and noisy conditions. (b) It offers simplicity as its training includes the relatively simple identification of an FP–AR model and the determination of the evolution function, while its operation in real time requires a simple optimization procedure and the validation of the tread estimate that directly leads to the wheels useful mileage through the evolution function. (c) The estimation of the wheel tread depth provides valuable insight about the evolution of the hollow wear over the running mileage and conditions such as abrupt and long braking, imbalanced payload, and so on.
(iii) The RULE results of the method’s assessment based on the four sets of signals from the corresponding re-profiling cycles in the inspection phase indicated high accuracy in tread depth estimation from very early stages of hollow wear with the maximum mean absolute error to be 0.075 mm (based on Set 3), while the remaining mileage maximum mean error of 8170 km corresponds to the 3.34% of the total mileage.
(iv) The results from the comparison with a multi-health state classification MM–AR-based method, involving 2040 inspection test cases, revealed the superior performance of the introduced method, although it is not designed to operate in such a classification framework. It achieved an overall True Positive Rate of 81.17% and precision of 82.51% for the considered health states, with its competitor to demonstrate poor performance with an overall True Positive Rate of 45.44% and precision of 45.66% .

Despite its various advantages, the main limitations of the study include the requirement of known samples of tread depths at corresponding mileage for a complete re-profiling cycle, and the potential decrease on the method’s accuracy for applications where the pattern of the evolution function significantly alters in different life cycles, yet this is not expected for railway vehicle wheels.

However, future work includes further exploration of the method’s performance using solely experimental data, a second accelerometer, as well as varying operating conditions such as different payload. Furthermore, the case of a varying pattern evolution function in subsequent re-profiling cycles along with the potential use of additional wheels critical dimensions (i.e., flange gradient, thickness, height) will be also investigated in order to extend and solidify the method as a valuable tool of high precision and simplicity for railway vehicle predictive maintenance.

## Figures and Tables

**Table 1:** Actual leading wheel tread depths at corresponding mileages based on an Attiko Metro S.A. vehicle.

Tread Depth (mm)	Running Mileage (km)
0.34	57,633
0.72	109,795
1.03	133,129
1.55	185,342
1.93	217,723
2.27	244,786

**Table 2:** Details on the considered HW conditions and vibration signals of the 566 Monte Carlo simulations.

No. of Tread Depths *k* (HW Conditions)	No. of Signals	
	Baseline Phase	Inspection Phase
137 with k∈ [0–2.3] mm	18	548 (4 sets of 137 signals *)
(mileage interval: 1800 km)	(mileage interval: 14,400 km)	
Sampling freq: $f_{s}=150$ Hz; Bandwidth: [0–75] Hz; signal length: *N* = 3600 samples;		
traveling speed: 35 m/s; Total running mileage: $\tau_{M}$ = 244,800 km ( M=18 )		

* Each set correspond to a different re-profiling cycle and contains signals from all available tread depths.

**Table 3:** Details on the baseline and inspection phases of the RULE method.

Baseline phase				
Estimated Model	No. of projection coefficients	Samples Per Parameter	Condition Number ^a^	BIC
FP–AR(62)_4_	248	261.29	$7.66\times 10^{4}$	−62.55
FP–AR: *Estimation via Ordinary Least Squares (OLS)* [21]; *No. of signals: M=18 ; Signal length: N=3600 samples *				
Functional subspace: Dimensionality determination via Genetic Algorithm, *Population = 100, Elite count = 20,*				
*Number of generations = 50, Function tolerance = $10^{-14}$ , Matlab function:* ga.m				
Functional basis *p*: *4 Shifted Legendre polynomials* $G=\{G_{0}\left(k\right),\;G_{1}\left(k\right),\;G_{2}\left(k\right),\;G_{3}\left(k\right)\}$ ^b^				
Evolution function: * $k=\overset{f(\tau )}{\overset{︷}{ae^{b\tau }-a}}$ with a=1.80 and $b=3.33\times 10^{-6}$ *, *Matlab function*: fit.m				
**Inspection phase**				
Wheel tread depth estimation: *Estimation of k via NLS estimator (golden search & parabolic interpolation), Function tolerance = $10^{-10}$ ,*				
*Step Tolerance = $10^{-10}$ , Matlab function:* fminbnd.m				
Validation: *Pena–Rodriguez residual whiteness test; user selected threshold Z=0.3 (No. of lags =100 )*				
Remaining Useful Life Estimation: *Based on Equation (4) is* ${\hat{\tau }}_{u}^{r}=\tau_{M}-b^{-1}\ln\frac{\hat{k}+a}{a}$				

^a^ Condition number of $\Phi^{T}\Phi$ (refer to Equation (11) in [21]); ^b^ $G_{n}$ : univariate orthogonal polynomial of degree *n*.

**Table 4:** Mean Absolute Error (MAE) for wheels tread depth and remaining mileage estimation per signal set.

Signal Set	MAE	
	Tread Depth	Remaining Mileage (%) *
1	0.074 mm	$8.17\times 10^{3}$ km ( 3.34% )
2	0.072 mm	$7.72\times 10^{3}$ km ( 3.15% )
3	0.075 mm	$8.09\times 10^{3}$ km ( 3.30% )
4	0.072 mm	$7.84\times 10^{3}$ km ( 3.20% )

* Percentage over total mileage $\tau_{M}$ = 244,800 km.

**Table 5:** Investigated health state classes of wheels with HW for classification-based RULE along with the corresponding ranges of tread depths and remaining mileages.

Health State Class	Tread Depth Range (mm)	Remaining Mileage Range (mm)
HW–1	[0, 0.22)	[244,800, 210,000)
HW–2	[0.22, 0.47)	[210,000, 175,000)
HW–3	[0.47, 0.74)	[175,000, 140,000)
HW–4	[0.74, 1.07)	[140,000, 105,000)
HW–5	[1.07, 1.42)	[105,000, 70,000)
HW–6	[1.42, 1.83)	[70,000, 35,000)
HW–7	[1.83, 2.30]	[35,000, 0]

**Table 6:** Total scores from multi-health classification-based RULE.

Method	True Positive Rate (TPR) (%)	Precision (PPV) (%)
FP–AR(62)_4_	81.17	82.51
MM–AR(62)	45.44	45.66

## Data Availability

No new data were created or analyzed in this study. Data sharing is not applicable to this article.

## Abbreviations

The following abbreviations are used in this manuscript:

AR	AutoRegressive
BIC	Bayesian Information Criterion
FP	Functionally Pooled
HW	Hollow Wear
MM	Multiple Model
OLS	Ordinary Least Squares
PSD	Power Spectral Density
RULE	Remaining Useful Life Estimation
